# CREPT/RPRD1B promotes tumorigenesis through STAT3-driven gene transcription in a p300-dependent manner

**DOI:** 10.1038/s41416-021-01269-1

**Published:** 2021-02-03

**Authors:** Wanli Zhai, Xiongjun Ye, Yinyin Wang, Yarui Feng, Ying Wang, Yuting Lin, Lidan Ding, Liu Yang, Xuning Wang, Yanshen Kuang, Xinyuan Fu, Y. Eugene Chin, Baoqing Jia, Bingtao Zhu, Fangli Ren, Zhijie Chang

**Affiliations:** 1 State Key Laboratory of Membrane Biology, School of Medicine, Tsinghua University, Beijing, China; 2 Tsinghua-Peking Joint Center for Life Sciences, School of Life Science, Tsinghua University, Beijing, China; 3 Urology and Lithotripsy Center, Peking University People’s Hospital, Beijing, China; 4 Department of General Surgery, Chinese PLA General Hospital, Beijing, China; 5 Laboratory of Human Diseases and Immunotherapies, West China Hospital, Sichuan University, Beijing, China; 6 Institutes of Biology and Medical Sciences, Soochow University, Suzhou, China

**Keywords:** Growth factor signalling, Transcriptional regulatory elements

## Abstract

**Background:**

Signal transducer and activator of transcription 3 (STAT3) has been shown to upregulate gene transcription during tumorigenesis. However, how STAT3 initiates transcription remains to be exploited. This study is to reveal the role of CREPT (cell cycle-related and elevated-expression protein in tumours, or RPRD1B) in promoting STAT3 transcriptional activity.

**Methods:**

BALB/c nude mice, CREPT overexpression or deletion cells were employed for the assay of tumour formation, chromatin immunoprecipitation, assay for transposase-accessible chromatin using sequencing.

**Results:**

We demonstrate that CREPT, a recently identified oncoprotein, enhances STAT3 transcriptional activity to promote tumorigenesis. CREPT expression is positively correlated with activation of STAT3 signalling in tumours. Deletion of CREPT led to a decrease, but overexpression of CREPT resulted in an increase, in STAT3-initiated tumour cell proliferation, colony formation and tumour growth. Mechanistically, CREPT interacts with phosphorylated STAT3 (p-STAT3) and facilitates p-STAT3 to recruit p300 to occupy at the promoters of STAT3-targeted genes. Therefore, CREPT and STAT3 coordinately facilitate p300-mediated acetylation of histone 3 (H3K18ac and H3K27ac), further augmenting RNA polymerase II recruitment. Accordingly, depletion of p300 abolished CREPT-enhanced STAT3 transcriptional activity.

**Conclusions:**

We propose that CREPT is a co-activator of STAT3 for recruiting p300. Our study provides an alternative strategy for the therapy of cancers related to STAT3.

## Background

Signal transducer and activator of transcription 3 (STAT3), a member of the STAT protein family, is constitutively activated in various tumours and promotes cancer progression and metastasis.^1–5^ STAT3 is a transcription factor that exists largely as a dimer formed by its amino-terminus domain in unstimulated cells. Upon the stimulation of cytokines or growth factors, STAT3 is phosphorylated at tyrosine 705 by tyrosine kinases (e.g. Janus-activated kinases 1, 2, and 3, or Src). After phosphorylation, the conformation of the pre-formed STAT3 dimer is stabilised by the interaction between the phosphotyrosine of one monomer and the SH2 domain of the other. Moreover, both unphosphorylated and phospho-Y705 STAT3 constitutively shuttles between the cytoplasmic and nuclear compartments. Reduced nuclear export due to DNA binding leads to nuclear accumulation of STAT3 in response to the cytokine.^6–8^ To date, it has been well documented that STAT3 regulates several genes related to cell proliferation (e.g. *c-MYC* and *CCND1*),^9,10^ cell survival (e.g. *Bcl-XL* and *BIRC5*),^11,12^ angiogenesis (e.g. *VEGF* and *HGF*)^2,13^ and inflammation responses (e.g. *IL-6* and *IL-10*).^14–16^ Upregulation of these genes eventually leads to cell cycle progression and resistance to apoptosis. Accordingly, normal cells with over-activated STAT3 gain the features of tumour cells.^5,10^ Reciprocally, elimination of STAT3 efficiently inhibits tumorigenesis and tumour cell growth in colon cancers.^17^ In addition, the expression and activation of STAT3 correlate with tumour grade, stage, the appearance of metastases and recurrence in breast cancers.^18^ To date, various STAT3 inhibitors have been developed for the therapy of different cancers.^19–21^ However, effective therapeutic strategies for inhibiting STAT3 remain to be further developed.^22^

The activity of STAT3 is precisely regulated in different cells. Both positive and negative factors have been identified to regulate the STAT3 activity. These factors include tyrosine kinases in the cytoplasm to boost the phosphorylation,^23–25^ phosphatases in both cytoplasm and nucleus to dephosphorylate STAT3^26,27^ and enzymes to regulate STAT3 acetylation and deacetylation.^28–30^ Among these factors, CBP/p300 has been reported to activate STAT3 by regulating transcription initiation. CBP/p300 mediates histone modification and facilitates chromatin remodelling by enhancing histone H3/H4 acetylation.^31–34^ The histone acetylation alteration eventually enriches the recruitment of RNA polymerase II (RNAPII) to transcript STAT3-targeted genes. However, how STAT3 initiates transcription remains to be exploited.

We previously identified a gene *CREPT* (cell cycle-related and elevated-expression protein in tumours, or *RPRD1B*) that is highly expressed in varieties of human tumours^35–44^ and also murine stem cells.^45^ CREPT promotes tumorigenesis through upregulation of a set of cell cycle-related genes. A mechanism study revealed that CREPT facilitates the recruitment of RNAPII to the promoter region and blocks its reading through the pol(A) signal in the termination region of the *CCND1* gene. CREPT promotes a loop formation of the *CCND1* gene to facilitate RNAPII redistribution to the promoter after termination^35^ and is found to physically interact with the C-terminal domain (CTD) of RNAPII.^35,46,47^ Recently, we found that p300 is a CREPT-interacting protein and CREPT increases the interaction between p300 and β-catenin to enhance the transcription of β-catenin/TCF4 in Wnt signalling.^48,49^ In the present study, we demonstrate that CREPT augments the STAT3 transcriptional activity through p300-mediated histone acetylation. CREPT and p300 cooperatively promote STAT3-driven tumorigenesis. We prospect that targeting both CREPT and STAT3 will be a promising intervention for cancer therapy.

## Methods

### Plasmids and reagents

Flag-CREPT, Myc-CREPT, Myc-CREPT/RPR, Myc-CREPT/CCT, Flag-STAT3, APRE (acute phase response element)-luciferase and pCDH-HA-CREPT were constructed in this laboratory. HA-p300 plasmid was a gift from Dr. Y. Eugene Chin (Institutes of Biology and Medical Sciences, Soochow University). Anti-STAT3 (c-20), anti-Myc (9E10) and anti-HA (F-7) were purchased from Santa Cruz Biotechnology (Santa Cruz). Anti-p300 (D2X6N), anti-c-MYC, anti-cyclinD1 (SP4), anti-pY705 STAT3 (D3A7), anti-pS727 STAT3, anti-acK685 STAT3, anti-Bcl-XL (54H6), anti-H3K18ac (D8Z5H) and anti-H3K27ac (D5E4) antibodies were purchased from Cell Signaling Technology. Anti-actin (AC-15) and anti-Flag (M2) antibodies were purchased from Sigma. Anti-CREPT antibody was prepared by this laboratory.^50^ The cytokine leukaemia inhibitory factor (LIF) was purchased from Millipore (cat. #LIF1010). Short interfering RNAs (siRNAs) against CREPT or p300 were synthesised from GenePharma (SuZhou GenePharma Co. Ltd) with the oligo sequence information as shown in Table S1. The CRISPR/Cas9 (clustered regularly interspaced short palindromic repeats/CRISPR-associated 9)-mediated CREPT deletion plasmids were generated based on PX458M vector with guider RNAs and the sequence information was shown in Table S1.

### Cell culture and transfection

HEK293T, NIH3T3 MOCK, NIH3T3 v-Src, MCF7 and SW480 cells were cultured in Dulbecco’s modified Eagle’s medium supplemented with 10% fetal bovine serum and penicillin/streptomycin. All the above cells were kept at 37 °C in a 5% CO_2_-containing atmosphere. Cells were transfected with plasmids as indicated using Vigofect (Vigorous Inc.), according to the manufacturer’s instruction. To generate stable cell lines, 3T3 MOCK cells or 3T3 v-Src-overexpressed cells were infected by a lentivirus, which was produced by HEK293T cells transiently transfected with vectors of pCDH and pLL3.7 carrying different complementary DNAs. Green fluorescent protein-positive cells were selected by fluorescence-activated cell sorting. For MCF7 or SW480 CREPT deletion cell lines, PX458 vector or PX458 carrying single guide RNAs for CREPT were transiently transfected into MCF7 or SW480 cells. Several single clones were randomly picked and identified by PCR.

### Luciferase assays

Indicated plasmids were co-transfected into the cells. After transfection for 24 h, cells were stimulated with LIF (20 ng/ml) or oncostatin M (OSM) (20 ng/ml) for 8 h. The reporter activity was examined by the Dual-Luciferase Assay System (Vigorous Inc.). Firefly luciferase activity was normalised against Renilla luciferase activity and presented as a mean ± standard deviation.

### Real-time quantitative PCR (QRT-PCR) analysis

Total RNA was extracted using TRIzol (Invitrogen). RNA was reversely transcribed using a Quantscript RT Kit (TIANGEN Biotech). QRT-PCR was performed using a Talent qPCR (quantitative polymerase chain reaction) PreMix (SYBR Green) Kit (TIANGEN Biotech) on a Roche machine using the following conditions: denature, 95 °C, 5 s; annealing, 60 °C, 10 s; and extension, 72 °C, 15 s. The sequence of the primers used for qRT-PCRs was shown in Table S2.

### Co-immunoprecipitation (co-IP)

Co-IP assay was performed as described.^4^ Specifically, for endogenous interaction assays, the nuclear fraction of SW480 or MCF7 cells was used for immunoprecipitation (IP).

### Chromatin immunoprecipitation (ChIP) assay

In brief, 1 × 10^7^ MCF7 cells in a 10-cm dish were treated with LIF for 30 min and fixed in 1% formaldehyde. Chromatin was sheared into 100–500 bp fragments with sonication. The lysates prepared from these cells were incubated with indicated antibodies for IP and ChIP analyses. Eluted DNA was used as templates for qPCR amplifications. The input control was from the supernatant before precipitation. The fragment corresponding to the APRE site was amplified by PCR with the primers shown in Table S3.

### Immunofluorescence staining

MCF7 cells were seeded on coverslips in a 6-well plate, incubated overnight at 37 °C and then transfected with the indicated plasmids. Cells were treated with LIF (20 ng/ml) for 30 min before fixation with 4% paraformaldehyde for 20 min and perforated with 0.3% Triton X-100 for 10 min. After blocking with 10% goat serum for 1 h at room temperature, cells were incubated with the indicated antibodies overnight at 4 °C, followed by incubation with the secondary antibodies conjugated with FITC (green) or TRITC antibody (Jackson Research Laboratories) for 1 h. Stained cells were visualised using a confocal laser scanning microscope (Olympus FV10iOil) with co-localisation of the two proteins indicated by a merged image.

### Experimental animals

Twelve 5-week-old female BALB/c nude mice in an SPF grade were employed to evaluate the in vivo effect of CREPT on STAT3-induced tumour growth. All mice were housed in isolated ventilated cages (maxima six mice per cage) barrier facility at Tsinghua University. The mice were maintained on a 12/12-h light/dark cycle, 22–26 °C with sterile pellet food and water ad libitum. The laboratory animal facility has been accredited by AAALAC (Association for Assessment and Accreditation of Laboratory Animal Care International) and the IACUC (Institutional Animal Care and Use Committee) of Tsinghua University approved all animal protocols used in this study.

### Tumour formation in the nude mice

A total of 2 × 10^5^ indicated cells were subcutaneously injected into the two flanks of the identical nude mice. Ten days after injection, mice were sacrificed by the euthanasia method of carbon dioxide inhalation. The volume of the tumours was measured by a ruler and the mass of the tumours was measured by an analytical balance.

### Colony formation assay

A total of 1 × 10^3^ indicated cells were seeded in 6-well plates with the treatment of LIF (20 ng/ml). After 2 weeks, the cells were stained with crystal violet. Statistical results are representative of three independent experiments.

### Assay for transposase-accessible chromatin using sequencing (ATAC-seq)

Briefly, 50,000 cells of each sample were used to generate DNA libraries for sequencing NextSeq500. All sequencing data were mapped on to hg19 using bowtie2. Peaks were called using MACS2.

### Statistical analysis

All experiments were repeated at least three times. Data were presented as mean ± standard deviation. Significant differences between the two groups were determined using the two-tailed Student’s *t* test.

## Results

### CREPT expression is positively correlated with STAT3 activation in human tumours

**Fig. 1:**
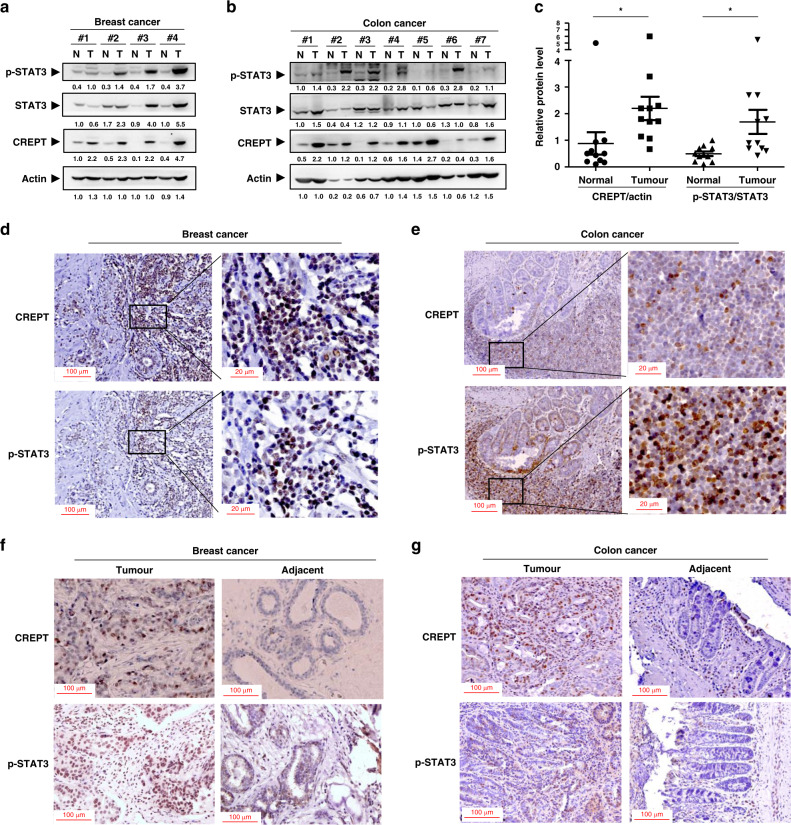
CREPT expression is positively correlated with STAT3 activation in human tumours. **a**, **b** The protein levels of CREPT and p-STAT3 in human breast cancers (**a**) and colon cancers (**b**). N refers to paired normal tissue and T refers to tumour tissue from the same patient. The bands were quantified using ImageJ and presented as a value normalised according to the moderate level of a band in each blot. **c** A graph presentation of CREPT/actin and p-STAT3/STAT3 in adjacent normal and tumour tissues. The grey values in **a**, **b** were used to calculate the ratio of CREPT/actin or p-STAT3/STAT3. Each sample was dotted by closed circle (normal tissue for CREPT/actin), square (tumor tissue for CREPT/actin), triangle (normal tissue for p-STAT3/STAT3) and inverted triangle (tumor tissue for p-STAT3/STAT3), and the average was subject to a statistic analysis between normal and tumour tissues (*p < 0.05). **d**, **e** Representative images for CREPT and p-STAT3 levels using immunohistochemical staining in breast (**d**) and colon (**e**) cancers with an antibody against CREPT or p-STAT3(Y705). **f**, **g** Representative images for CREPT and p-STAT3 in breast (**f**) and colon (**g**) tumour tissue and adjacent normal tissue.

To analyse the potential relevance of CREPT and STAT3 activation, western blot analyses were performed in human breast cancer (Fig. 1a) and colon cancer tissues (Fig. 1b). We quantified the level of the proteins and calculated the ratio of phosphorylated STAT3 (p-STAT3), an activated form of STAT3 phosphorylated at Y705, to total STAT3 to indicate the activation of STAT3. A statistical analysis showed that both levels of CREPT and STAT3 activation were higher in the tumour tissue than those in the paired adjacent non-tumour tissue (Fig. 1c). Furthermore, immunohistochemistry (IHC) analyses showed that CREPT and p-STAT3 were both strongly stained in the nucleus in breast and colon cancer tissues (Fig. 1d, e). To confirm the specificity of the antibody used for IHC, we stained two consecutive slices using CREPT (Fig. S1A) or p-STAT3 (Fig. S1B) antibody and an isotype control IgG. We observed a negative staining by both mouse and rabbit IgG, indicating the specificity of CREPT and p-STAT3 antibody. In addition, we used the adjacent normal tissue to compare with the tumour tissue for the correlation of CREPT and p-STAT3. Consistently, the tumour tissues exhibited higher levels of CREPT and p-STAT3 than adjacent normal tissues both in breast (Fig. 1f) and colon (Fig. 1g) cancer. The upregulation of CREPT and p-STAT3 in colon cancer tissue compared with the normal tissue was significantly correlated (*R* = 0.71, *p* = 0.038) (Fig. S1E). All these results suggest that the activation of STAT3 occurred simultaneously with elevated expression of CREPT in tumours.

To examine whether elevated CREPT level correlates to the expression of STAT3-downstream genes, we analysed the messenger RNA (mRNA) levels of several key genes including *c-MYC*, *CCND1* and *Bcl-XL* in breast and colon cancers from The Cancer Genome Atlas RNA-sequencing database. The results showed that in breast cancer, the expression of *CREPT* was positively correlated with *Bcl-XL* and weakly correlated with *c-MYC and CCND1* at the mRNA level (Fig. S1C). However, in colon cancer, CREPT exhibited a positive correlation with those three genes (Fig. S1D). These results suggest that CREPT might participate in the regulation of STAT3-activated gene expression.

### CREPT promotes tumorigenesis induced by activated STAT3

**Fig. 2:**
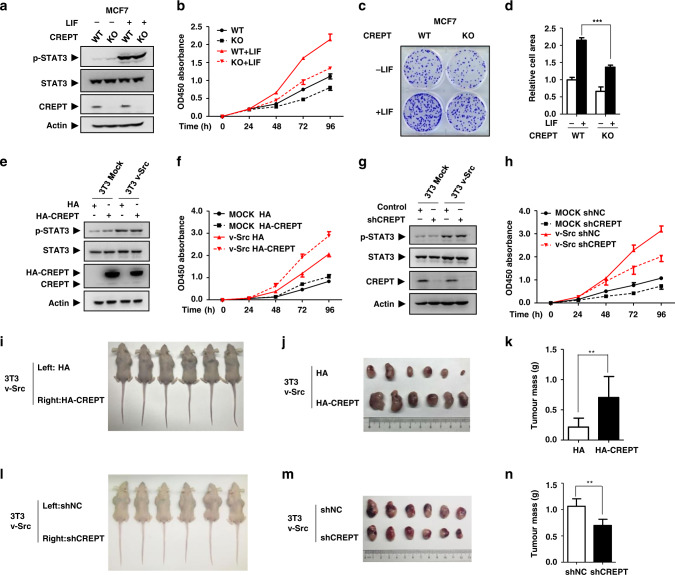
CREPT promotes tumorigenesis induced by activated STAT3. **a**–**d** Deletion of CREPT reduced LIF-induced cell proliferation (**a**, **b**) or colony formation (**c**, **d**) in MCF7 cells. **a** Establishment of CREPT deletion and its control cell lines based on MCF7 using CRISPR/cas9 system. The cells were further treated with or without the stimulation of LIF (20 ng/ml). **b** A cell proliferation assay was performed using CCK8 Kit. The absorbance at 450 nm was used as an indicator of cell numbers. **c**, **d** Colony formation assays were performed in MCF7 cells (***p < 0.001). **e** Establishment of cell lines with CREPT overexpression in v-Src-transformed and control 3T3 cells. **f** Overexpression of CREPT promotes the cell growth of v-Src-transformed cells. **g** Establishment of cell lines with CREPT depletion in v-Src-transformed and control 3T3 cells. **h** Depletion of CREPT inhibited v-Src-transformed cell proliferation. **i**–**k** Overexpression of CREPT promotes the tumour formation from v-Src-transformed cells. Tumour formation assay was performed in nude mice (**p < 0.01). **l**–**n** Depletion of CREPT reduced the tumour formation in the v-Src-transformed cells (**p < 0.01).

To reveal whether CREPT plays a role in the STAT3-induced tumorigenesis, a cell proliferation assay was performed in MCF7 cells under stimulation of LIF (leukaemia inhibitory factor), a cytokine that activates STAT3^51–55^ (Fig. 2a). The result demonstrated that deletion of CREPT inhibited the proliferation of the cells under normal condition (WT vs. KO; Fig. 2b). Interestingly, this inhibitory role of CREPT deletion became more obvious in the presence of LIF (WT + LIF vs. KO + LIF; Fig. 2b), indicating that CREPT facilitates LIF-induced cell proliferation. In addition, a colony formation assay indicated that the colony numbers formed by MCF7 cells were significantly decreased in response to LIF when CREPT was deleted (Fig. 2c, d). Similar results were observed in SW480 cells (Fig. S2A–D). To further address the role of CREPT in STAT3-induced tumours, we transformed NIH3T3 fibroblasts using v-Src, which constitutively induces STAT3 phosphorylation,^56^ in the presence of stably expressed HA-CREPT (Fig. 2e). A cell proliferation assay showed that HA-CREPT promoted the cell proliferation slightly in the control cells, but dramatically in the v-Src-transformed cells (Fig. 2f). Conversely, stable depletion of CREPT by a short hairpin RNA (Fig. 2g) significantly suppressed the proliferation of v-Src-transformed cells (Fig. 2h). These results suggest that CREPT could enhance STAT3 activity in promoting cell proliferation. To examine whether CREPT could promote STAT3-induced tumour formation, v-Src-transformed 3T3 cells were subcutaneously injected into the two flanks of the identical nude mice. The results showed that the tumours formed by v-Src-transformed cells with overexpression of HA-CREPT were significantly larger than those formed by control cells both in mass and volume (Fig. 2i–k). Furthermore, the tumours formed by CREPT depletion cells showed a smaller volume and a less mass (Fig. 2l–n). Taken together, all the results suggest that CREPT enhances the STAT3-induced tumorigenesis.

### CREPT enhances STAT3 transcriptional activity and the targeted gene expression

**Fig. 3:**
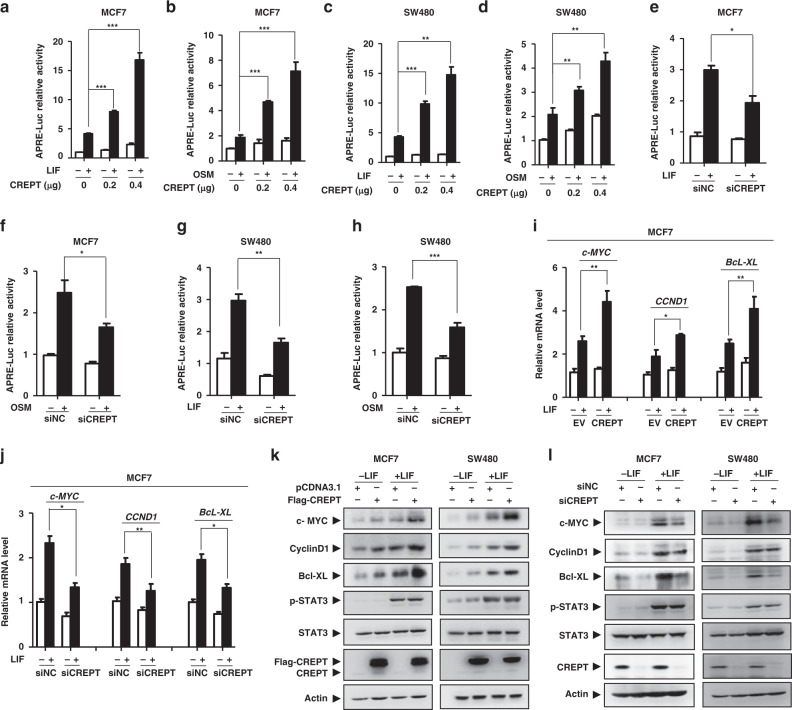
CREPT enhances the STAT3 transcriptional activity and the expression of STAT3-targeted genes. **a**–**d** Overexpression of CREPT promotes the APRE-luciferase activity. Luciferase assay was performed in wild-type or CREPT transiently overexpressing cells in the absence or presence of LIF (20 ng/ml) (**p < 0.01; ***p < 0.001). **e**–**h** Depletion of CREPT using a mixture of siRNA#1 and siRNA#2 resulted in a decreased transcriptional activity of STAT3 (*p < 0.05; **p < 0.01; ***p < 0.001). **i** CREPT promotes the expression of STAT3-targeted genes. Flag-CREPT plasmid was transfected into MCF7 cells with or without LIF (20 ng/ml) for 4 h. The mRNA levels of STAT3-targeted genes were examined by qRT-PCR. **j** Depletion of CREPT using a mixture of siRNA#1 and siRNA#2 impairs the expression of STAT3-targeted genes in response to LIF. **k** Overexpression of CREPT enhanced the protein levels of STAT3-targeted genes. Flag-CREPT plasmid was transfected into MCF7 (left) or SW480 (right) cells with or without LIF (20 ng/ml) for 12 h. The protein levels of STAT3-targeted genes were examined by Western blots. **l** Depletion of CREPT decreased the protein levels of STAT3-targeted genes.

To examine whether CREPT could regulate the STAT3 transcriptional activity, we performed a luciferase assay using the APRE reporter.^36,57^ Consistent with previous reports,^51,55,58–60^ LIF and OSM, two STAT3-activating cytokines, stimulated the STAT3 transcriptional activity, whereas overexpression of CREPT further enhanced the luciferase activity (Fig. 3a, b) in MCF7 cells. Consistently, overexpression of CREPT significantly enhanced the STAT3 transcriptional activity in SW480 (Fig. 3c, d), HEK293T (Fig. S3A) and HeLa (Fig. S3B) cells. These results suggest that CREPT could enhance the transcription activity of STAT3. To reveal the role of endogenous CREPT in STAT3-driven gene transcription, we performed a luciferase assay using specific siRNAs against CREPT. The results demonstrated that depletion of endogenous CREPT impaired the activation of luciferase in response to LIF in MCF7 (Fig. 3e, f), SW480 (Fig. 3g, h), HEK293T (Fig. S3C) and HeLa cells (Fig. S3D). These results suggest that CREPT is involved in the STAT3-driven transcriptional process. Furthermore, we found that STAT3-targeted genes including *CCND1*, *c-MYC* and *Bcl-XL* were upregulated by overexpression of CREPT in response to LIF in MCF7 and SW480 cells at mRNA (Fig. 3i and Fig. S3E) and protein (Fig. 3k) levels. To address the role of endogenous CREPT in the regulation of STAT3-driven genes, we depleted CREPT in MCF7 cells using siRNAs. The results showed that depletion of CREPT significantly reduced the levels of STAT3-targeted genes in response to LIF at both mRNA (Fig. 3j and Fig. S3F) and protein (Fig. 3l) levels. Taken together, these results suggest that CREPT is a potent regulator for the STAT3 transcriptional activity.

### CREPT preferentially interacts with p-STAT3 via the RPR domain

**Fig. 4:**
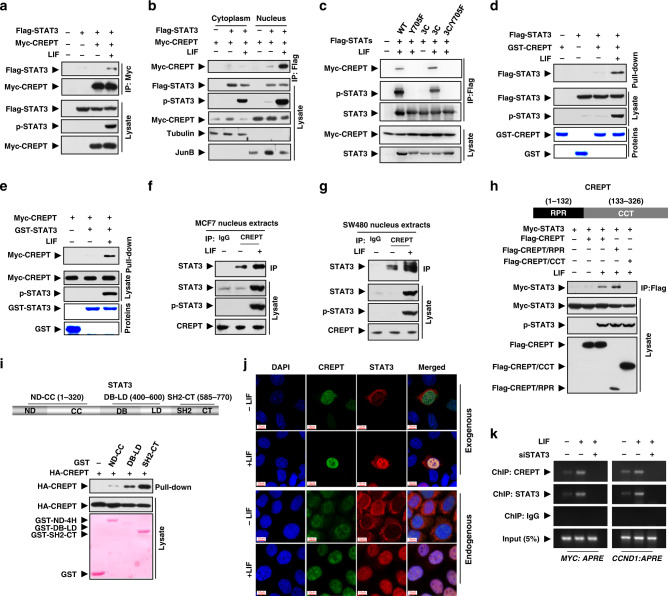
CREPT preferentially interacts with p-STAT3 via its RPR domain. **a** Myc-CREPT interacts with Flag-STAT3 in response to LIF. **b** The interaction of CREPT and STAT3 occurs in the nucleus. The nuclear marker JunB and the cytoplasmic marker tubulin were used to demonstrate the purity of fractions. **c** Interaction of CREPT with STAT3 depends on STAT3 phosphorylation rather than its nuclear localisation. **d**, **e** CREPT interacts with STAT3 physically. A GST pull-down assay was performed with purified GST or GST-CREPT protein and Flag-STAT3 from HEK293T cell lysates (**d**). A reciprocal GST pull-down assay was performed with purified GST or GST-STAT3 protein and Flag-CREPT from HEK293T cell lysates (**e**). **f**, **g** Endogenous interaction between CREPT and STAT3. Nuclear extracts from MCF7 (**f**) or SW480 (**g**) cells were immunoprecipitated with an anti-CREPT antibody. **h** The RPR domain, but not CCT domain of CREPT interacts with Myc-STAT3. i CREPT preferentially interacts with the SH2-CT domain of STAT3. **j** CREPT co-localises with STAT3 upon LIF treatment. MCF7 cells were treated with LIF (20 ng/ml) or a control medium for 30 min, followed with immunostaining using an anti-CREPT antibody and an anti-STAT3 antibody. **k** CREPT and STAT3 co-occupied at the promoter region of STAT3-targeted genes. ChIP assay was performed with the CREPT or STAT3 antibody in MCF7 cells with or without the treatment of LIF (20 ng/ml) for 30 min.

To reveal how CREPT promotes the transcriptional activity of STAT3, we addressed whether CREPT could interact with STAT3. An IP experiment using exogenously expressed Flag-STAT3 and Myc-CREPT was performed in HEK293T cells. The result showed that Myc-CREPT precipitated down Flag-STAT3 in the presence of LIF, whereas no interaction was observed without LIF (Fig. 4a), suggesting that CREPT and STAT3 interact under the condition of LIF stimulation. Another co-IP experiment using cytoplasmic and nuclear fractions revealed that the interaction between STAT3 and CREPT occurred in the nucleus and was augmented by LIF (Fig. 4b). Since LIF induces STAT3 phosphorylation and nuclear translocation, we questioned whether the interaction of CREPT with STAT3 depends on the phosphorylation or the nuclear location of STAT3, as CREPT always locates in the nucleus.^35^ We took advantage of STAT3C, a mutant that constitutively localises in the nucleus, STAT3Y705F, a mutant that lost the phosphorylation,^61^ and STAT3C/Y705F, a double mutant that lost the phosphorylation but remains to localise in the nucleus as a dimer.^36^ An IP experiment showed that STAT3Y705F failed to interact with CREPT in the presence of LIF, whereas STAT3C interacted with CREPT only in the presence of LIF (Fig. 4c). Furthermore, STAT3C/Y705F, which localises in the nucleus but is not phosphorylated, failed to interact with CREPT in the presence of LIF (Fig. 4c, last lane). These results suggest that the interaction of CREPT and STAT3 depends on the phosphorylation status of STAT3.

To address whether CREPT physically binds to p-STAT3, we performed a glutathione *S*-transferase (GST) pull-down experiment with GST-CREPT purified from *Escherichia coli* and Flag-STAT3 purified from HEK293T cells under LIF stimulation. The result showed that GST-CREPT pulled down LIF-stimulated Flag-STAT3 but failed to associate with untreated Flag-STAT3 (Fig. 4d). Reciprocally, we observed that the purified GST-STAT3 protein under LIF stimulation pulled down Myc-CREPT, but untreated GST-STAT3 failed to interact with Myc-CREPT in vitro (Fig. 4e). Obviously, p-STAT3 was detected in the LIF-stimulated GST-STAT3 but not in the untreated GST-STAT3 (Fig. 4e). The results suggest that CREPT interacts with p-STAT3. Consistent with these in vitro results, the interaction between endogenous CREPT and STAT3 was also observed by IP experiments with an antibody against CREPT in the nuclear extracts of MCF7 (Fig. 4f) and SW480 (Fig. 4g) cells. Significantly, LIF enhanced the interaction of CREPT and STAT3 in the nucleus of both cells (Fig. 4f, g). Taken together, we conclude that CREPT interacts directly with p-STAT3.

To analyse the detailed interaction structure of CREPT and p-STAT3, we generated deletion mutants with different domains of CREPT (Fig. 4h, top) and STAT3 (Fig. 4I, top). An IP experiment showed that the RPR domain, but not the CCT domain of CREPT, interacts with STAT3 (Fig. 4h). Another GST pull-down experiment using purified proteins showed that CREPT preferentially interacted with the SH2-CT domain and the DB-LD domain of STAT3 (Fig. 4i). Furthermore, we performed an immunostaining assay to investigate the co-localisation of CREPT with STAT3 in MCF7 cells. The results showed that CREPT co-localised with STAT3 in the nucleus after LIF treatment (Fig. 4j). Furthermore, we observed that the occupancy of CREPT on promoters of STAT3-targeted genes (*c-MYC* and *CCND1*) was enhanced by LIF stimulation and depletion of STAT3 abolished the CREPT occupancy (Fig. 4k). Our results suggest that STAT3 mediates the occupancy of CREPT at promoters of STAT3-targeted genes. Collectively, all the results demonstrated that CREPT preferentially interacts with p-STAT3 to regulate STAT3 transcriptional activity.

### CREPT augments histone H3 acetylation through enhancing p300 occupancy on the promoter of STAT3-targeted genes

To further decipher how CREPT facilitates the STAT3 transcriptional activity, we examined the possible influence of CREPT on STAT3 phosphorylation, acetylation and occupancy on the promoters of its targeted genes. Western blot analyses demonstrated that overexpression (Fig. S4A) or depletion (Fig. S4B) of CREPT had no effect on the levels of p-STAT3 (Y705), p-STAT3 (S727) and ac-STAT3 (K685), three major active forms of STAT3,^30,62^ in different cell lines under LIF stimulation. In addition, a ChIP assay with primers flanking the acute phase binding element (APRE, specific for STAT3 binding) in the *c-MYC* or *CCND1* gene demonstrated that STAT3 bound to APRE of the *c-MYC* or *CCND1* promoter was little altered in cells with overexpression or deletion of CREPT (Fig. S4C, D). These results suggest that the enhanced transcriptional activity of STAT3 by CREPT is due to neither the increase in the STAT3 modifications nor its occupancy to the promoter region of the downstream target genes.

**Fig. 5:**
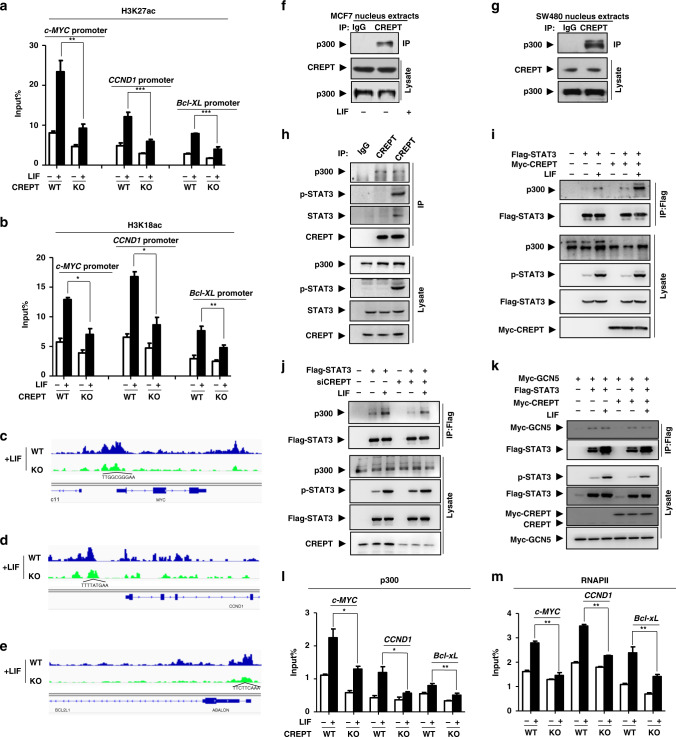
CREPT augments the level of acetylated histone H3 through enhancing p300 occupancy on the promoter of STAT3-targeted genes. **a**, **b** Deletion of CREPT decreases the level of ac-H3K27 (**a**) or ac-H3K18 (**b**) on the APRE region. **c**–**e** Loss of CREPT decreases chromatin accessibility at the promoter region of STAT3. ATAC-seq peaks at the promoter regions of the STAT3-targeted genes from wild-type (blue) or CREPT depletion (green) MCF7 cells were shown. The STAT3 binding consensus sequence was labelled at the corresponding locus. **f**, **g** The endogenous interaction between CREPT and p300. Nuclear extracts from MCF7 (**f**) or SW480 (**g**) cells were immunoprecipitated with an anti-CREPT antibody. **h** CREPT, STAT3 and p300 formed a ternary complex in MCF7 cells. Cell lysates were precipitated by an anti-CREPT antibody. **i**, **j** CREPT enhances the interaction of STAT3 and p300. Flag-STAT3 was co-expressed with Myc-CREPT (**I**) or siRNAs against endogenous CREPT (**j**) in MCF7 cells. **k** Overexpression of CREPT hardly affected the interaction between STAT3 and GCN5. **l**, **m** Deletion of CREPT attenuates p300 (**l**) or RNAPII (**m**) occupancy on the promoters of STAT3-targeted genes. The quantity indicating the presence of p300 (**l**) or RNAPII (**m**) on APRE was normalised with respective inputs. (*p < 0.05; **p < 0.01; ***p < 0.001).

We further postulated that CREPT might shape the active enhancer landscape in STAT3 binding region, as enhancers are known to be the major determinants of specific gene expression.^63^ Therefore, the active enhancer markers H3K27ac and H3K18ac in the promoters of STAT3-targeted genes were examined. Intriguingly, the quantitative ChIP-qPCR result showed that deletion of CREPT significantly decreased the levels of H3K27ac and H3K18ac at the APRE region of *c-MYC*, *CCND1* and *Bcl-XL* genes (Fig. 5a, b). Reciprocally, overexpression of CREPT significantly enhanced the abundance of H3K27ac and H3K18ac (Fig. S5A, B). Accordingly, an ATAC-seq result showed that loss of CREPT significantly decreased the chromatin accessibility at the promoter region of STAT3-targeted genes (Fig. 5c–e). These results suggest that CREPT elevated the active modifications of histone 3 flanking the STAT3 binding region. We, therefore, conclude that CREPT promotes histone modifications H3K27ac and H3K18ac to enhance the STAT3-driven gene transcription.

To reveal the mechanism how CREPT elevates the histone acetylation, we determined to investigate whether p300, a canonical histone acetyltransferase, is involved in CREPT-enhanced modification of histone 3, as p300 was isolated in the CREPT-interacting complex by our mass spectrum analysis.^48^ The association of endogenous CREPT with p300 was verified in MCF7 (Fig. 5f) and SW480 cells (Fig. 5g). Intriguingly, we observed that CREPT, p-STAT3 and p300 formed a ternary complex in MCF7 cells under LIF stimulation (Fig. 5h). To examine whether CREPT could influence the association of p300 with STAT3, a co-IP experiment was performed. The results showed that the association of p300 with STAT3 was dramatically increased in CREPT overexpression cells (Fig. 5i, lane 3 vs. 6), but was obviously decreased in CREPT depletion cells (Fig. 5j, lane 3 vs. 6). However, overexpression of CREPT failed to affect the interaction of STAT3 with GCN5, another canonical acetyltransferase (Fig. 5k). These results suggest that CREPT specifically enhances the interaction of STAT3 with p300 under LIF stimulation. Furthermore, we observed that deletion of CREPT significantly attenuated p300 occupancy to the APRE of the *c-MYC*, *CCND1* and *Bcl-XL* promoters (Fig. 5l), suggesting that CREPT increases the association of p300 to the promoters of STAT3-targeted genes. Accordingly, the occupancy of RNAPII was also impaired in CREPT deletion cells (Fig. 5m). Taken together, all the results indicate that CREPT strengthens the p300 occupancy on the APRE region of STAT3-downstream genes and augments the histone 3 acetylation, resulting in enhanced accessibility of RNAPII.

### p300 is required for CREPT-enhanced STAT3 transcriptional activity

**Fig. 6:**
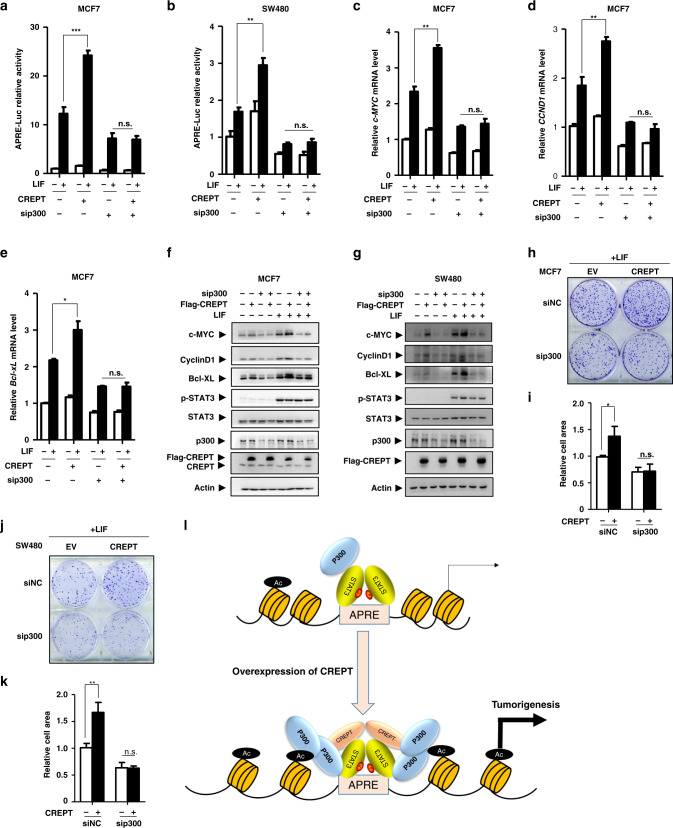
p300 is required for CREPT-enhanced STAT3 transcriptional activity. **a**, **b** Depletion of p300 impaired the enhanced effect of CREPT on STAT3 transcriptional activity. **c**–**g** Depletion of p300 impaired the STAT3-targeted gene expression promoted by CREPT both at mRNA (**c**–**e**) and protein (**f**, **g**) levels examined by QRT-PCR and western blot. **h**–**k** CREPT promotes colony formation dependent on p300. Colony formation assays were performed in MCF7 (**h**, **i**) or SW480 (**j**, **k**) cells. A representative colony is shown in (**h**, **J**) and the quantitative numbers are shown in (**i**, **k**). **l** A model indicating that highly expressed CREPT in tumour cells facilitates the recruitment of p300 to the promoter of STAT3-targeted genes (*p < 0.05; **p < 0.01; ***p < 0.001; n.s., no statistical difference).

To further verify the effect of p300 on CREPT-enhanced STAT3 transcription activation, a STAT3-specific luciferase reporter assay was performed. The results showed that overexpression of CREPT significantly enhanced the luciferase activity, but depletion of p300 abolished its effect in MCF7 (Fig. 6a) or SW480 (Fig. 6b) cells. These results suggest that p300 is required for CREPT in promoting STAT3-driven gene transcription. To investigate the physiological effect of p300 and CREPT on gene transcription, we examined the expression of *CCND1*, *c-MYC* and *Bcl-XL*, three major STAT3-targeted genes. The results showed that CREPT failed to promote these gene expressions in the absence of p300 at both the mRNA (Fig. 6c–e) and protein (Fig. 6f, g) levels. Consistently, a colony formation assay showed that CREPT promoted the cell proliferation in the presence of p300, but failed when p300 was depleted in both MCF7 (Fig. 6h, k) cells. Taken together, these data suggest that p300 is an essential factor for the CREPT-enhanced STAT3 transcriptional activity.

## Discussion

STAT3 has been demonstrated to play multiple roles in cell growth, cell survival and tumour immunity through activating the transcription of various genes including *c-MYC*, *CCND1* and *Bcl-XL*.^9–11^ STAT3-driven transcription requires several cofactors, such as CBP/p300, NcoA/SRC1a, and hCTR9. These factors induce modifications on histone tails, coupled with chromatin remodelling, thereby enhancing target gene expression. In the present study, we identified CREPT as a novel cofactor to regulate STAT3 transcriptional activity through p300. We proposed that CREPT bridges the interaction of STAT3 and p300 and promotes the histone acetylation for the accessibility of the chromatin (Fig. 6l). We have provided a set of evidence based on ATAC-seq analysis, gene expression patterns in the patients from The Cancer Genome Atlas database, biochemistry analyses and tumour models from mice. Our results from both human samples and mouse models suggest that CREPT regulated STAT3 activation is critical for tumorigenesis. This study extends the knowledge on underlying mechanisms of the positive regulation of STAT3 transcriptional activity by co-activators during tumorigenesis.

An important question addressed in the present study is the molecular basis for the regulation of STAT3 transcriptional activity by CREPT. The kinases of STAT3 (Src and JAK) are localised in the cytoplasm, while CREPT is localised in the nucleus. We have observed that both the canonically activated forms of STAT3, p-STAT3(Y705)^6–8,48^ and p-STAT3(S727), another well-known phospho-site of STAT3, essential for the maximal activation of STAT3,^62^ were unaffected by CREPT. Moreover, the occupancy of p-STAT3 on the promoter region was hardly affected by CREPT. These features are analogous to previous findings on the co-activator NcoA/SRC1a, which enhances STAT3 transcriptional activity without affecting its phosphorylation or occupancy.^33^ On the other hand, it is possible that CREPT expression and the activation of STAT3 could be associated in tumours. Indeed, we observed elevated CREPT is correlated with p-STAT3. The simultaneous occurrence of elevated expression of CREPT and the activation of STAT3 provides the basis for CREPT to interact with activated STAT3, enhancing its transcriptional activity through other factors. Interestingly, we observed that CREPT, p300 and p-STAT3 form a ternary complex, which enhances the enrichment of histone acetylation markers H3K18ac and H3K27ac flanking STAT3 binding region. However, CREPT fails to affect the acetylation of STAT3 proteins. This is a surprising discovery as STAT3 has been widely reported as a substrate of p300.^30,64^ Indeed, our experiment confirmed that LIF enhances the acetylation of STAT3. However, it appears that CREPT has no effect on LIF-induced acetylation of STAT3 (Fig. S4A, B). Because the interaction of CREPT with STAT3 is highly related to LIF stimulation but the interaction of CREPT with p300 seemed constitutive, we propose that it is p-STAT3, after its binding at the promoter, to recruit the CREPT/p300 complex to acetylate histone 3. This mechanism is similar to the function of CREPT on the regulation Wnt/β-catenin pathway.^48^ Similar mechanism was reported on the regulation of histone modification by p53 with p300 recruitment.^65^ Our study provides a general model for the histone modification by p300 through transcriptional factors, where CREPT functions as an adaptor to bridge the association of transcriptional factors, p300 and histones (Fig. 6l).

CREPT has no typical DNA-binding domain, but indeed occupies on the promoter region of selected genes.^35,39,49^ Our results showed that LIF stimulation increases the occupancy of CREPT on STAT3-targeted gene promoters. We propose that it is STAT3 that selects the DNA sequences at the promoter to recruit CREPT occupancy. We speculate that other transcriptional factors might also recruit CREPT to specific transcriptional sites. As CREPT also interests with RNAPII,^35,46,66–68^ we consider that sequence-specific transcriptional factors recruit CREPT, which mediates the recruitment of RNAPII at the transcription initiation. In this study, we confirmed our hypothesis by providing an enhanced complex formation of CREPT/p300 to promote RNAPII occupancy directed by STAT3 into specific gene promoters such as *CCND1*, *c-MYC* and *Bcl-XL*. Furthermore, we will identify the elaborate binding site for STAT3 in CREPT by truncation-mediated immunoprecipitation assay. A small-molecule inhibitor designed by the binding site could disturb the interaction between CREPT and STAT3, thus interfering the development of STAT3-related tumours. In addition, as the advance of PROTAC in targeting CREPT,^69^ we expect that targeting the interaction of CREPT and STAT3, for instance, the SH2-CT fragment, will be more efficient to inhibit tumour growth.

In conclusion, we propose that STAT3 recruits CREPT to associate with p300 to acetylate histone 3 at the promoters of STAT3-targeted genes. This is critical for the oncogene transcription during tumorigenesis. We prospect that targeting CREPT can be used to develop synthetic inhibitors for STAT3-related tumours.

## Supplementary information

FigureS1-S5

Sequence information for the siRNAs and the guider RNAs

Sequence information for primers used for qRT-PCR

Sequence information for primers for ChIP-qPCR

## Data Availability

Raw data and materials generated during the present study are available from the corresponding author upon reasonable request.
