# Supplementary material for: CREPT/RPRD1B promotes tumorigenesis through STAT3-driven gene transcription in a p300-dependent manner
Source: Br J Cancer. 2021 Feb 3;124(8):1437–48. doi: 10.1038/s41416-021-01269-1 (PMC8039031; doi:10.1038/s41416-021-01269-1)
Supplement: Supplementary file 1 — FigureS1-S5 [file 41416_2021_1269_MOESM1_ESM.pdf]

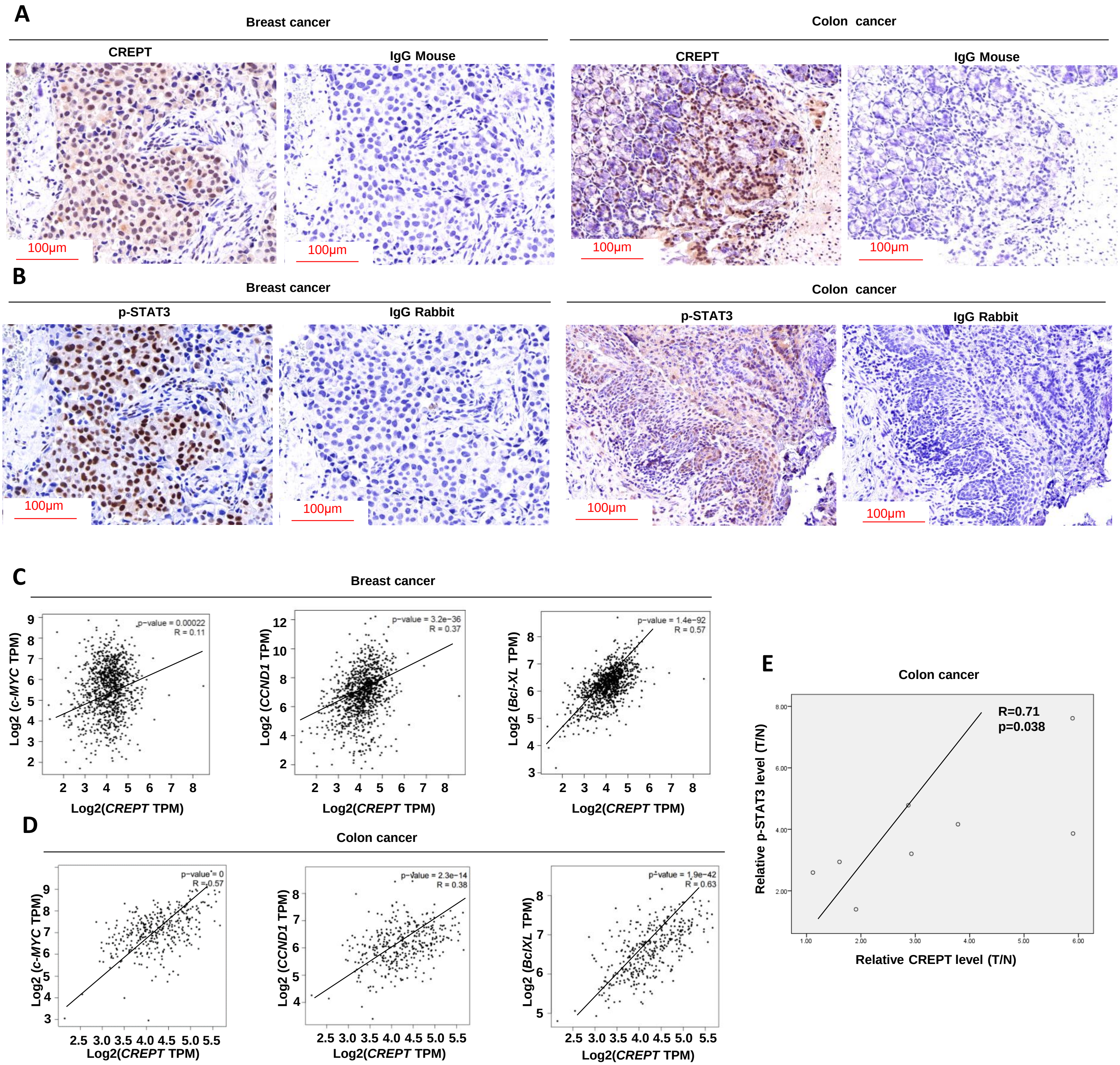

S1. CREPT expression is positively correlated with STAT3 activation in human tumors.

(A-B) Representative images for CREPT (A) or p-STAT3 (B) and the isotype control on a serial section using immunohistochemical staining in breast (left) and colon (right) cancers with indicated antibodies.

(C-D) Correlation of CREPT expression with STAT3-targeted genes. A plot dot presentation showed the correlation of expression of CREPT and STAT3-targeted genes in breast (C) and colon (D) cancer (original data was from http://gepia.cancer-pku.cn/).

(E) A graph presentation of correlation of CREPT and p-STAT3 in colon cancer. The level of CREPT and p-STAT3 in tumor tissue and the adjacent normal tissue were quantified using Image J. The ratio of CREPT (X-axis) or p-STAT3 (Y-axis) level in tumor tissue to the adjacent normal tissue was calculated. SPSS was employed to figure out the correlation coefficient between the two proteins. T refers to tumor, N refers to adjacent normal tissue.

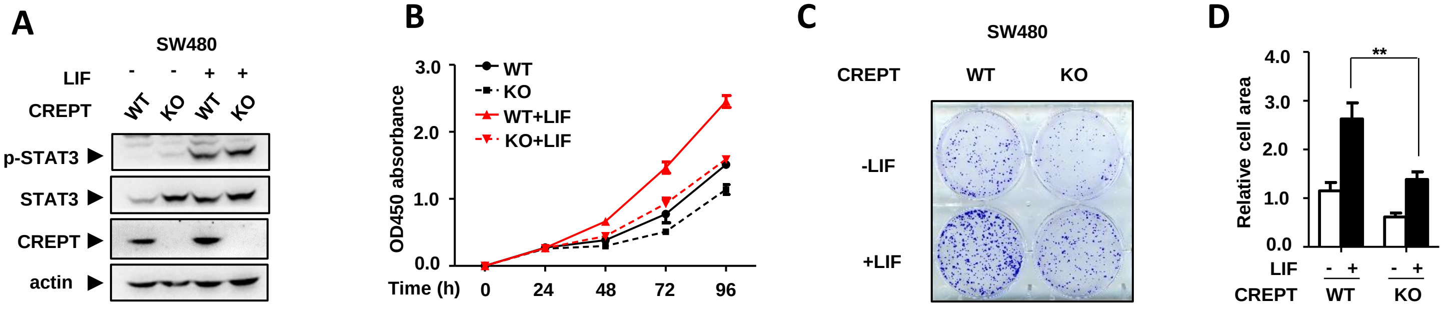

S2. CREPT promotes tumorigenesis induced by activated STAT3.

(A-D) Deletion of CREPT reduced LIF-induced cell proliferation (A-B) or colony formation (C-D) in SW480 cells. (A) Establishment of CREPT deletion and its control SW480 cell lines using CRISPR/cas9 system. (B) A cell proliferation assay was performed using CCK8 kit. A total of $1x10^3$ indicated cells were seeded in 96-well plates with or without the treatment of LIF (20ng/ml). (C-D) Colony formation assays were performed in SW480 cells. A total of $1x10^3$ indicated cells were seeded in 6-well plates with or without the treatment of LIF (20ng/ml). A representative colony is shown in (C) and the quantitative numbers are shown in (D) (**, $p < 0.01$).

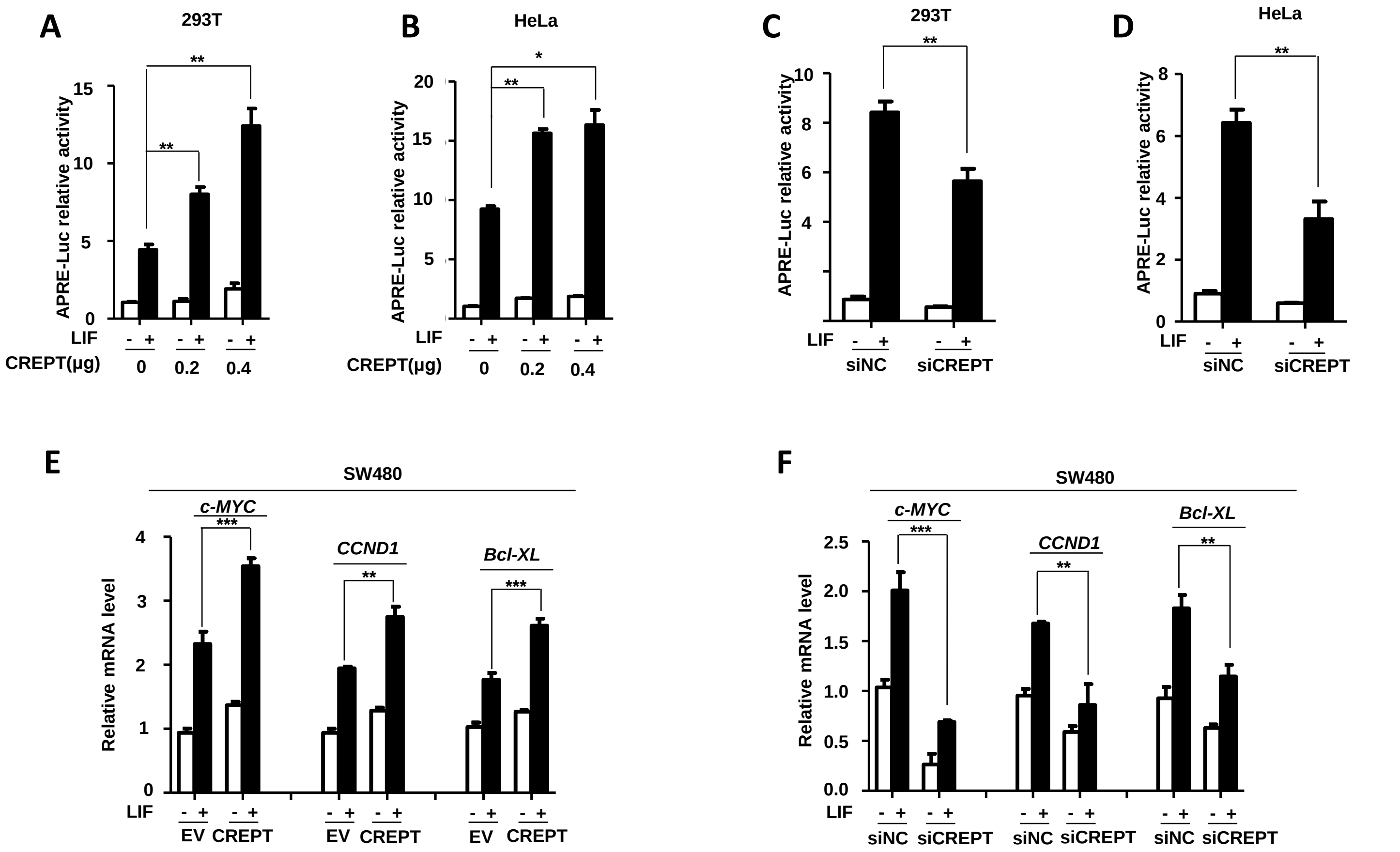

S3. CREPT enhances STAT3 transcriptional activity and the expression of STAT3-targeted genes.
(A-B) Overexpression of CREPT promotes the APRE-luciferase activity. STAT3 specific APRE-luciferase reporter and pRL-TK plasmids were co-transfected into 293T (A) or HeLa (B) cells in the presence of different dosages of the Flag-CREPT plasmids. After transfection for 24 h, cells were stimulated with LIF (20 ng/ml) for 8 h. The luciferase activity was expressed as fold-changes, normalized by an internal control (Renilla). Results were from three independent repeats and are presented as mean ± S.D. (*, $p < 0.05$; **, $p < 0.01$). (C-D) Depletion of CREPT resulted in a decreased transcriptional activity of STAT3. 293T cells (C) or HeLa cells (D) were transfected with an siRNA mixture against CREPT. The cells were re-seeded and further transfected with the APRE-luciferase reporter and pRL-TK plasmids. The luciferase activity was examined as in (A-B). A nonspecific siRNA (siNC) was used as a control. (**, $p < 0.01$). (E) CREPT promotes the expression of STAT3-targeted genes. Flag-CREPT plasmid was transfected into SW480 cells with or without the treatment of LIF (20 ng/ml) for 4 h. The mRNA levels of STAT3-targeted genes were examined by RT-qPCR . (F) Depletion of CREPT impairs the expression of STAT3-targeted genes in response to LIF. SW480 cells were transfected with an siRNA mixture against CREPT (siCREPT) and a nonspecific siRNA (siNC) as a negative control. Cells were treated with or without LIF (20 ng/ml) for 4 h. The mRNA levels of CCND1, c-MYC, Bcl-XL were examined by RT-qPCR.

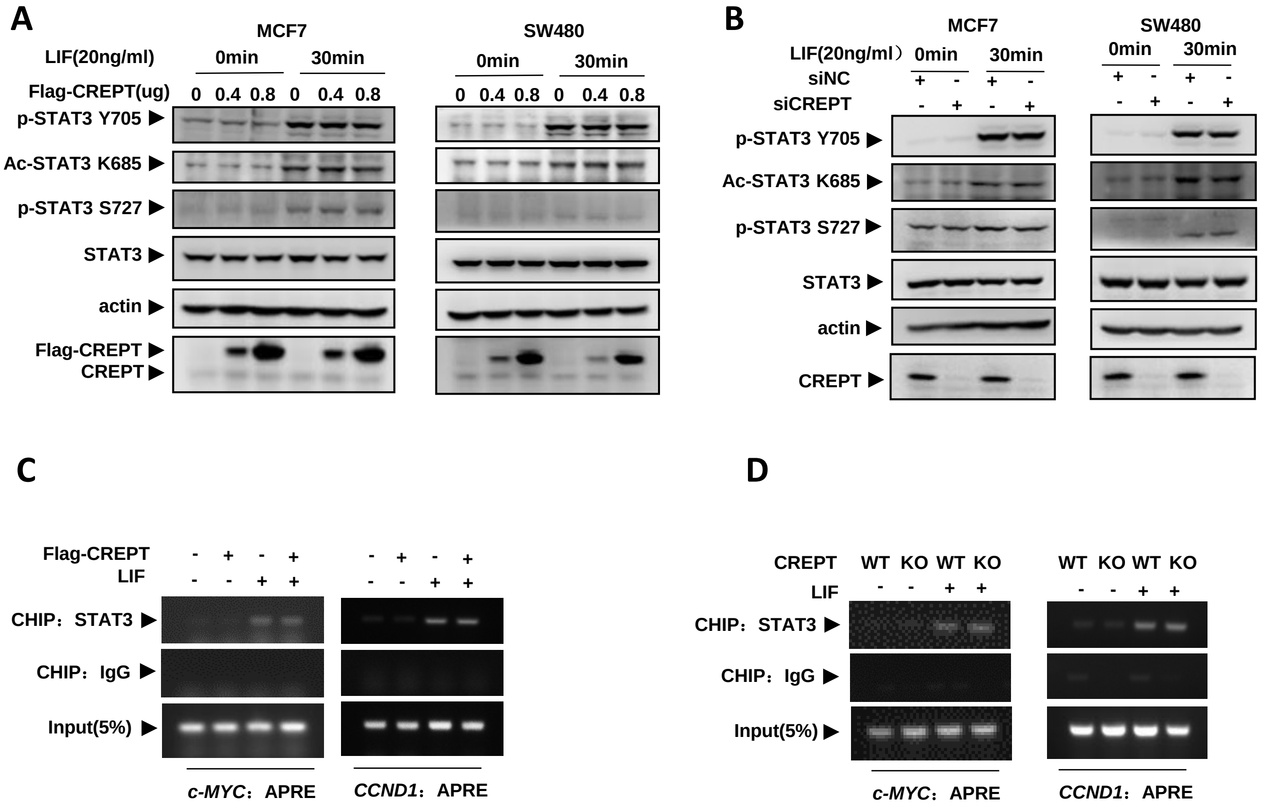

S4. CREPT fails to influence the levels of p-STAT3 (Y705, S727) and ac-STAT3 (K685).

(A) Overexpression of CREPT hardly affected the levels of STAT3 activated forms. Flag-CREPT plasmid was transfected into MCF7 (left) or SW480 (right) cells with or without the treatment of LIF (20 ng/ml) for 30 min. The levels of STAT3 activated forms were examined by Western blots. (B) Knockdown of CREPT hardly affected the levels of STAT3 activated forms. MCF7 (left) or SW480 (right) cells were transfected with siRNA mixture against CREPT (siCREPT) and a nonspecific siRNA (siNC) as a negative control. Cells were treated with or without LIF (20 ng/ml) for 30 min. The levels of STAT3 activated forms were examined by Western blots.(C) Overexpression of CREPT hardly affected STAT3 occupancy to the promoter region of c-MYC gene (left) and CCND1 gene (right). MCF-7 cells were transfected with Flag-CREPT or pCDNA3.1-Flag plasmids. After transfection for 24 h, the cells were treated with or without LIF for 30 min. ChIP assay was performed using an anti-STAT3 antibody. Gel base was applied to show the amounts of ChIPed DNA. （D）Knockout of CREPT hardly affected STAT3 occupancy to the promoter region of c-MYC gene (left) and CCND1 gene (right) . ChIP assay was performed using an anti-STAT3 antibody in wildtype or CREPT deletion MCF7 cells. The cells were treated with or without LIF for 30 min before harvest. Gel base was applied to show the amounts of ChIPed DNA.

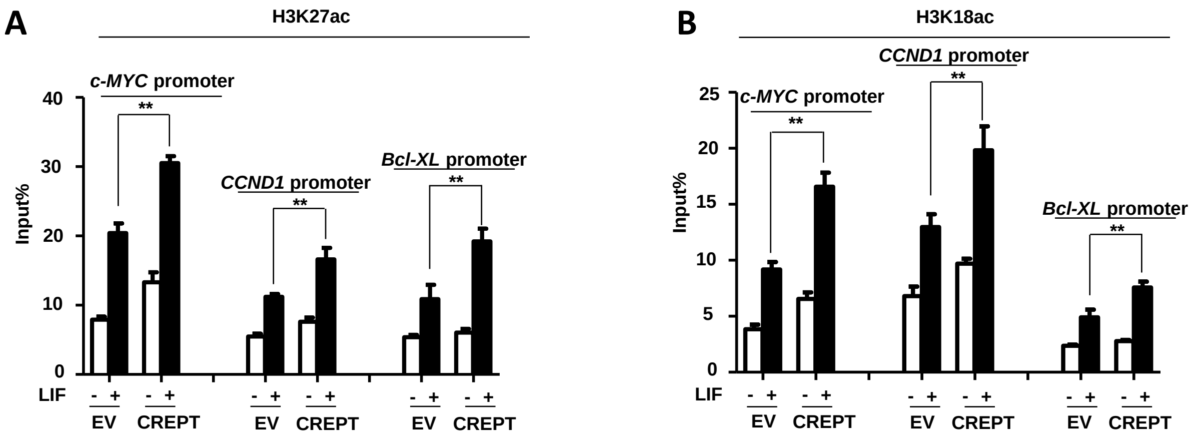

S5. CREPT augments the level of acetylated histone H3 through enhancing p300 occupancy on the promoter of STAT3-targeted genes.

(A-B) Overexpression of CREPT increases the level of ac-H3K27 (A) or ac-H3K18 (B) on the APRE. Flag-CREPT plasmid was transfected into MCF-7 cells, which were further treated with or without LIF (20ng/ml) for 30 min. A ChIP assay was performed using an anti-ac-H3K27 (A) or anti-ac-H3K18 (B) antibody. RT-qPCR was applied to quantify the amounts of ChIPed DNA, which were normalized with the input (**, p <0.01).
